# Pathological complete response by advanced hepatocellular carcinoma with massive macrovascular invasion to hepatic arterial infusion chemotherapy: a case report

**DOI:** 10.1186/s12957-019-1772-8

**Published:** 2019-12-26

**Authors:** Shusei Sano, Shinji Nakata, Shuichi Wada, Masatsugu Kuroiwa, Hiroki Sakai, Kei Kusama, Taiichi Machida, Akihito Nishio, Ichiro Ito, Harutsugu Sodeyama

**Affiliations:** 1 Department of Gastroenterological Surgery, Nagano Red Cross Hospital, 5-22-1, Wakasato, Nagano-shi, Nagano, 380-8582 Japan; 2 Department of Gastroenterology, Nagano Red Cross Hospital, Nagano, Japan; 3 Department of Pathology, Nagano Red Cross Hospital, Nagano, Japan

**Keywords:** Hepatocellular carcinoma, Macrovascular invasion, Hepatic arterial infusion chemotherapy, Complete response

## Abstract

**Background:**

Advanced hepatocellular carcinoma (HCC) with macrovascular invasion has an extremely dismal prognosis. We report a rare case of multiple HCC with tumor thrombosis in the portal vein and inferior vena cava that was initially treated with hepatic arterial infusion chemotherapy (HAIC); later resection revealed pathological complete response.

**Case presentation:**

A 75-year-old man presented with HCC in his right liver, with tumor thrombosis growing to the right portal vein and the inferior vena cava, and bilateral intrahepatic liver metastases. He underwent HAIC (5-fluorouracil [170 mg/m^2^] + cisplatin [7 mg/m^2^]) via an indwelling port. Although the tumor shrank and tumor marker levels decreased rapidly, we abandoned HAIC after one cycle because of cytopenia. We resumed HAIC 18 months later because of tumor progression, using biweekly 5-fluorouracil only [1000 mg] due to renal dysfunction. However, after 54 months, the HAIC indwelling port was occluded. The patient therefore underwent a right hepatectomy to resect the residual lesion. Histopathological findings showed complete necrosis with no viable tumor cells. The patient has been doing well without postoperative adjuvant therapy for more than 10 years after initially introducing HAIC and 6 years after the resection, without evidence of tumor recurrence.

**Conclusions:**

HAIC can be an effective alternative treatment for advanced HCC with macrovascular invasion.

## Background

Advanced hepatocellular carcinoma (HCC) with macrovascular invasion has an extremely poor prognosis, with a reported median survival time (MST) of 2.7–3.1 months if left untreated [1, 2]. In global guidelines, HCC with portal vein tumor thrombosis (PVTT) or inferior vena cava tumor thrombosis (IVCTT) is classified as the advanced stage, for which only systemic chemotherapy is recommended, even in patients with good liver function [3, 4]. Sorafenib is the standard of care for Child–Pugh A advanced HCC with macrovascular invasion and/or extrahepatic metastasis, and it significantly improved overall survival compared with supportive care [4–6]. However, MST for patients with sorafenib-treated HCC with macrovascular invasion is still poor, reportedly only 8.1 months [7]. Lenvatinib was recently shown to be non-inferior to sorafenib as first-line treatment [8], but therapeutic options are very limited for advanced HCC.

In Eastern Asian countries, various treatment options are available for HCC with macrovascular invasion, including systemic chemotherapy, hepatic arterial infusion chemotherapy (HAIC), transcatheter arterial chemoembolization, and surgery. Treatments are selected individually, depending on the extent of tumor thrombosis, degree of underlying cirrhosis, and patient’s performance status, which can affect prognosis. However, no guidelines clarify a preferred non-operative treatment based on available evidence. We herein report a patient with multiple HCC with PVTT and IVCTT who survived after HAIC, followed by a resection that showed pathological complete response (CR) and tumor-free status for more than 6 years.

## Case presentation

**Fig. 1:**
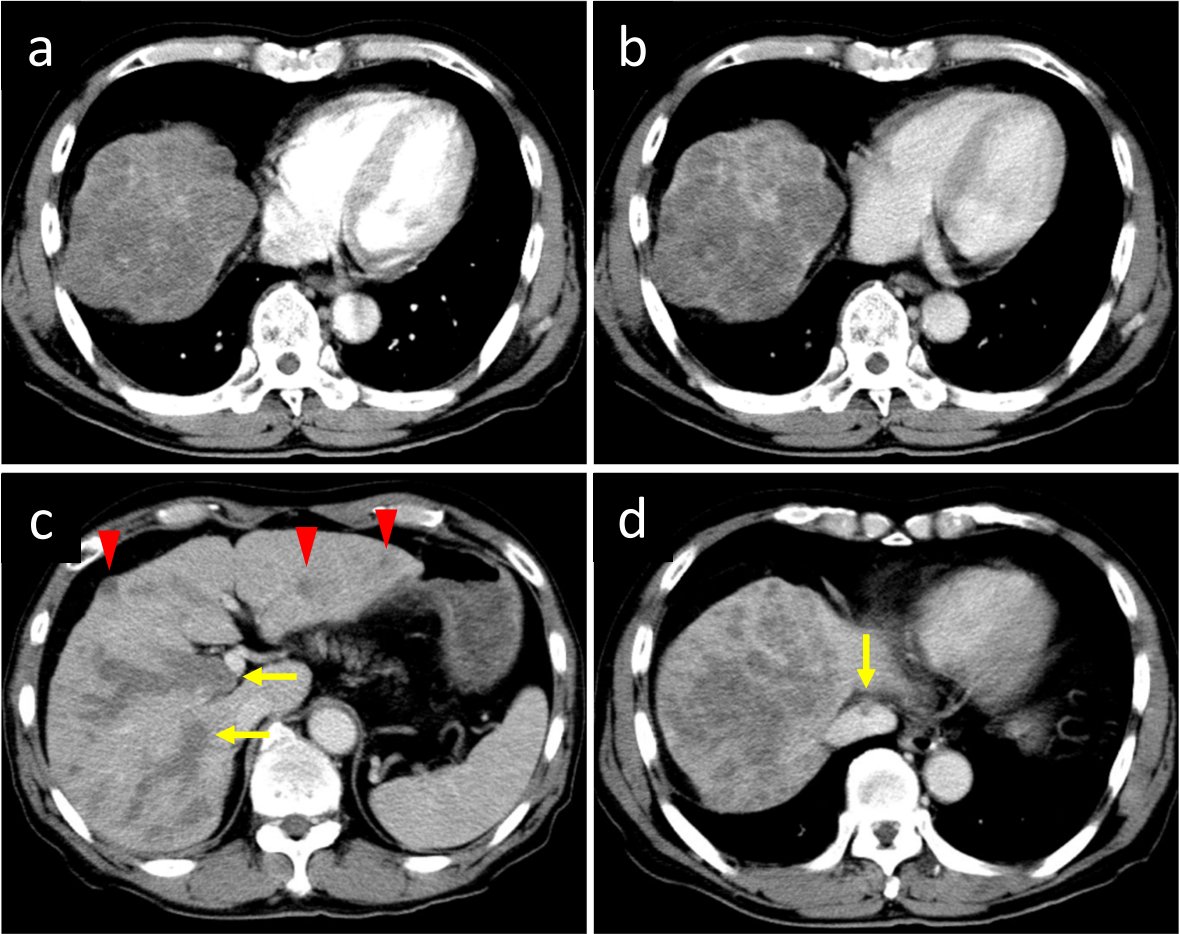
Enhanced CT images before introducing HAIC. Heterogeneous, 13-cm mass in the right liver shows hypovascular appearance in the arterial phase (**a**) and in the portal phase (**b**). Massive tumor thrombosis (arrow) growing to the right portal vein (**c**) and inferior vena cava (**d**), with bilateral intrahepatic liver metastases (arrowhead) (**c**). CT, computed tomography; HAIC, hepatic arterial infusion chemotherapy

In February 2009, a 75-year-old man with a history of alcoholic liver disease was referred to our hospital for evaluation of multiple liver masses on abdominal ultrasound sonography. The patient had no history of hepatitis of B or C infection. Abdominal enhanced computed tomography (CT) showed 13-cm hypovascular liver tumors (Fig. 1a, b), with marked tumor thrombosis growing to the right portal vein (Fig. 1c) and inferior vena cava (Fig. 1d), and bilateral intrahepatic liver metastases (Fig. 1c). Serum alpha-fetoprotein (AFP) level and protein induced by vitamin K absence or antagonist-II (PIVKA-II), also known as des-gamma-carboxyprothrombin, level were 3565 ng/ml and 49,000 mAU/ml, respectively. Chest CT scan, upper gastrointestinal endoscopy, and colonoscopy showed no other tumors. His carcinoembryonic antigen and carbohydrate antigen 19-9 levels were in the normal range.

**Fig. 2:**
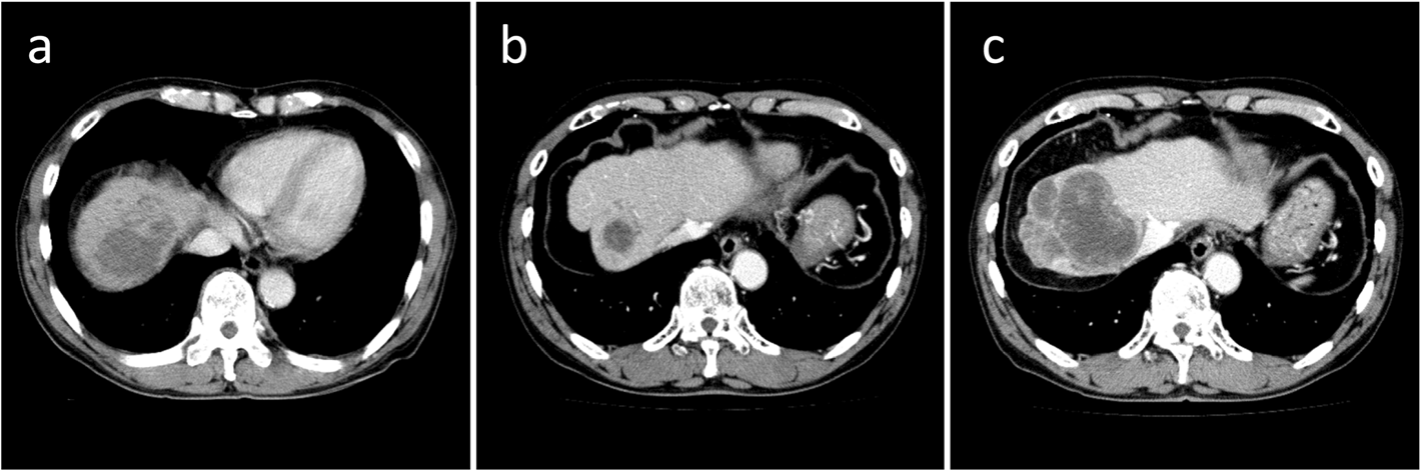
Enhanced CT images after introducing HAIC. Remarkable regression of intrahepatic lesions, thrombosis in the right portal vein and inferior vena cava is seen after one HAIC cycle (**a**). Tumor remained shrunk without treatment for 16 months after interrupting HAIC (**b**). Tumor progression occurred 18 months after the HAIC interruption (**c**)

**Fig. 3:**
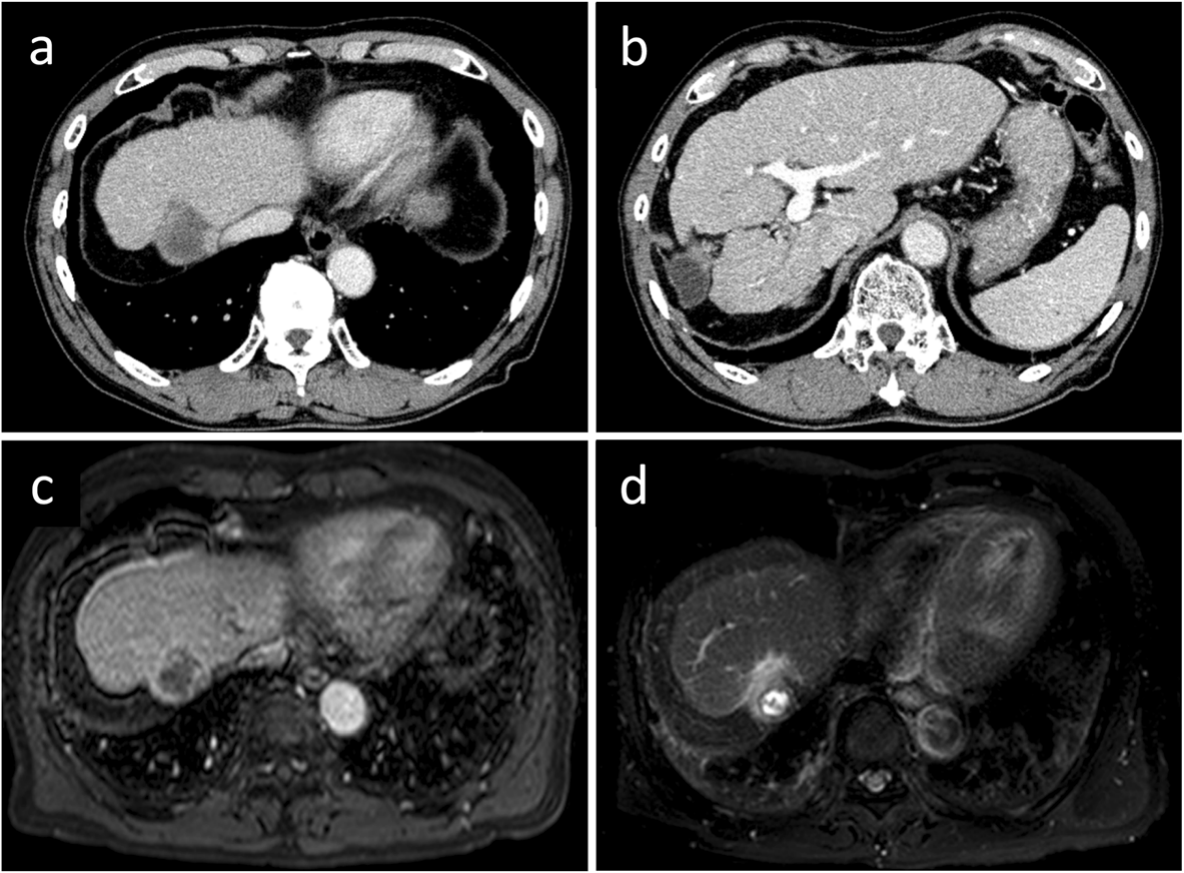
Enhanced CT and magnetic resonance imaging images before hepatectomy (54 months after the initial HAIC). Persistent tumor shrinkage with necrotic area, and no tumor thrombosis in the portal vein or the inferior vena cava, or intrahepatic metastases (**a**, **b**). Intratumoral necrotic area in the portal phase (**c**) and diffusion-weighted imaging (**d**)

Under the diagnosis of unresectable advanced HCC, an indwelling port was inserted, and HAIC with 5-fluorouracil (5-FU, 170 mg/m^2^) and cisplatin (7 mg/m^2^) continuously on days 1–5 via an implanted catheter system was administered. One cycle of HAIC consisted of 5 days of treatment and 2 days rest per week for 4 consecutive weeks. Despite significant decrease in tumor markers and remarkable regression of intrahepatic lesions, PVTT, and IVCTT on enhanced CT after one HAIC cycle (Fig. 2a), we abandoned this treatment due to leukopenia and thrombocytopenia. Eight months later, when his AFP elevated to 202 ng/ml, the patient refused our recommendation of sorafenib, which had become available in Japan at that year. After 18 months, during which the tumor remained silent and he was followed closely without treatment (Fig. 2b), his AFP and PIVKA-II levels rapidly elevated to 21,490 ng/ml and 1444 mAU/ml (respectively), and enhanced CT showed tumor progression (Fig. 2c). Therefore, we resumed the HAIC at the same dose for one cycle, but switched to 5-FU alone (1000 mg biweekly) due to renal dysfunction. Twenty-one months after resuming HAIC, we stopped this treatment because the indwelling port became occluded. At that time, the patient’s serum AFP and PIVKA-II were within normal ranges, and enhanced CT and magnetic resonance images indicated that the tumor was still shrunken with necrotic areas, and showed no PVTT, IVCTT, or intrahepatic metastases (Fig. 3). He had good hepatic function (Child–Pugh classification A5 and liver damage A) with atrophy of the right hepatic lobe (131 ml, corresponding to 15.1% of liver volume), despite indocyanine green retention rate being 15.0%. We therefore performed a right hepatic lobectomy to remove the residual lesion, at 54 months after his initial treatment. He was discharged on postoperative day 14 without postoperative complications.

**Fig. 4:**
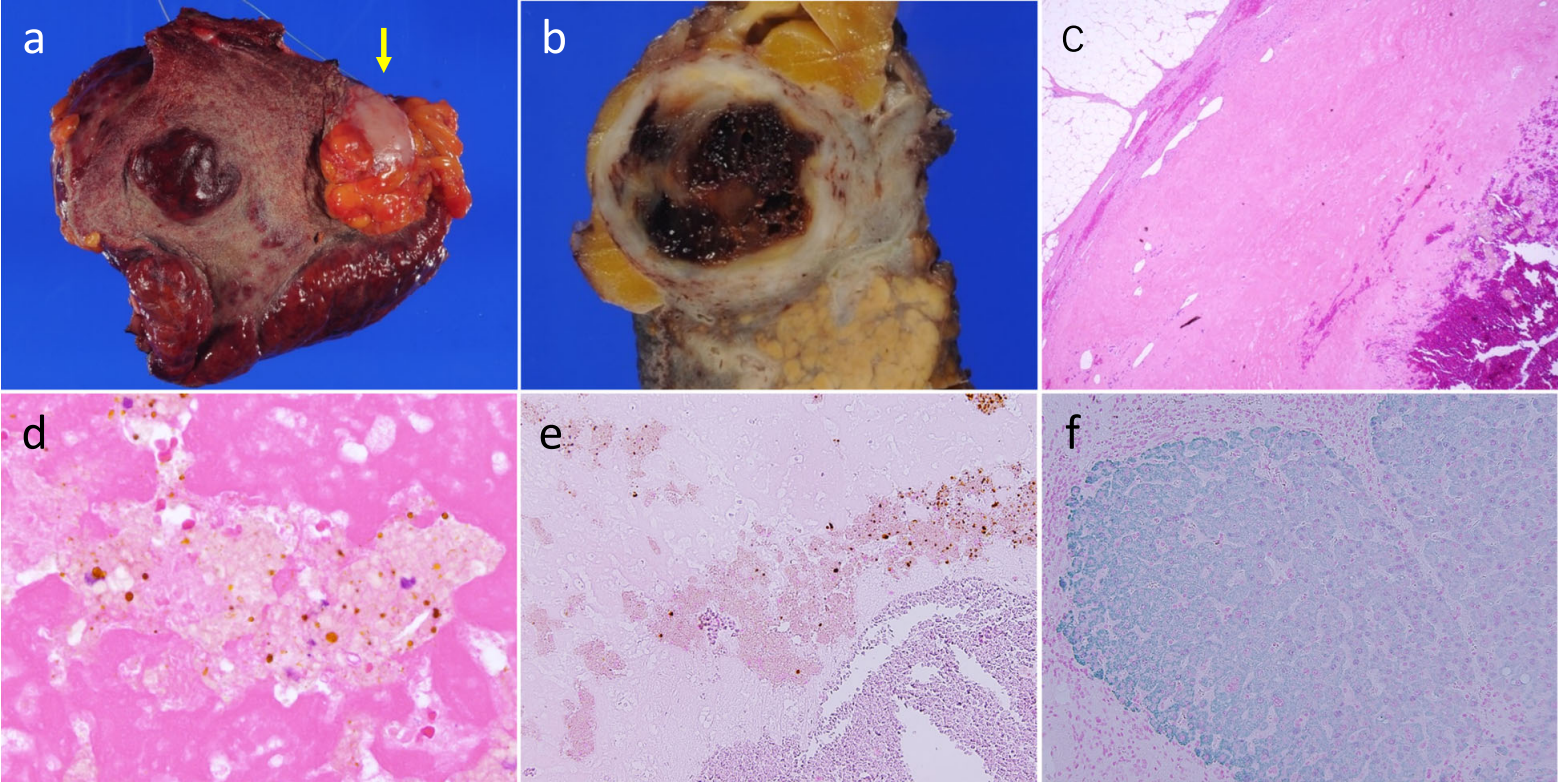
Gross and histopathological findings of the resected specimen. Whitish tumor surrounded by omentum at the liver surface (arrow). Background liver was composed of geographical atrophic area and cirrhotic liver parenchyma (**a**). The cut surface of the tumor shows the solid tumor with significant hemorrhage and necrosis (**b**). Microscopic finding of the liver mass shows complete necrosis surrounded by a thick hyalinized fibrotic capsule without any viable tumor cells (hematoxylin–eosin staining, × 40) (**c**). Bilirubin pigments surrounded by necrotic tissue in the central necrotic area (hematoxylin–eosin staining, × 400) (**d**). Immunohistochemical staining with hepatocyte specific antigen antibody shows nucleated cells in the tumor are negatively stained (× 200) (**e**), while green dye positive on non-tumorous hepatocyte (× 200) (**f**)

**Fig. 5:**
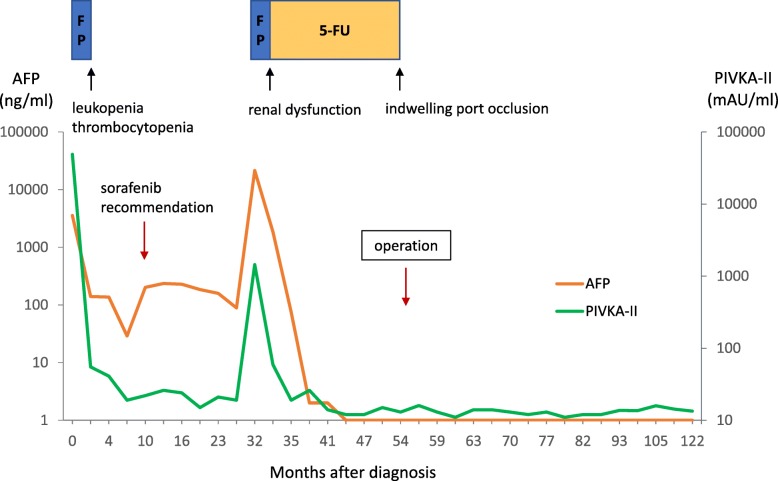
Clinical course as shown by tumor markers, therapeutic events, and adverse event. Tumor markers are displayed by logarithmic scale. 5-FU, 5-fluorouracil; AFP, alpha-fetoprotein; FP, 5-fluorouracil and cisplatin; HAIC, hepatic arterial infusion chemotherapy; PIVKA-II, protein induced by vitamin K absence or antagonist-II

The resected specimen showed the solid tumor with significant hemorrhage and necrosis (Fig. 4a, b). Microscopic examination revealed a nodule with a central necrotic core, surrounded by a thick hyalinized fibrotic capsule (Fig. 4c, d). No residual viable tumor cells were observed (Fig. 4d, e). Bilirubin pigments surrounded by necrotic tissue in the central necrotic compartment indicated that the tumor was HCC (Fig. 4d). No adjuvant therapy was performed. CT imaging has shown no signs of recurrence, and his tumor markers have also been within the normal limits for the past 126 and 72 months after the initial HAIC and after the operation, respectively. His clinical course is summarized in Fig. 5.

## Discussion

The present case shows the effectiveness of HAIC for advanced HCC with multiple intrahepatic metastases, PVTT, and IVCTT. A CR was pathologically proven after conversion surgery. We believe that HAIC would be the main cause of complete remission in this patient because of the following reasons: First, the patient did not receive medical care other than HAIC. Second, tumor regression completely coincided with the timing of HAIC. Tumor shrinkage and decrease in tumor markers were observed only when he received HAIC. Although CR in advanced HCC patients with macrovascular invasion has been previously described, most of these cases were treated with sorafenib alone [9, 10] or sorafenib combined therapy [11–16]. Only four reports written in English have shown a CR from HAIC in patients with advanced HCC [17–20]. Therefore, the present case was a rare case of CR achieved by HAIC alone, leading to a curative surgical resection following overall survival of more than 6 years without any adjuvant treatment.

HAIC uses high concentrations of anticancer agents administered directly into the hepatic artery via an injection port. It can enhance efficacy of drugs by localizing their application and minimizing systemic adverse effects. HAIC is frequently used against advanced HCC with macrovascular invasion in Eastern Asian patients. Among several HAIC protocols, the combination of 5-FU and cisplatin is one of the most common therapeutic regimens although there are slight differences of dose and duration setting in each study; a high response rate of 31–48% and improved MST of 14.0–31.6 months have been reported [2, 21–24]. Several studies have shown the efficacy of HAIC compared with sorafenib for advanced HCC with macrovascular invasion. Moriguchi et al., in a study of severe tumor thrombus in the first branches of the portal vein and/or the main portal vein, found MST (10.1 vs. 3.9 months) and median time-to-treatment-failure (3.5 vs. 1.2 months) were significantly longer in the HAIC with 5-FU and cisplatin group than in the sorafenib group [24]. Nakano et al. reported a prospective cohort study in which the therapeutic response rate of HAIC using cisplatin suspension in Lipiodol combined with 5-FU (New FP) was superior to that of sorafenib; median overall survival for the New FP and sorafenib groups was 30.4 and 13.2 months, respectively (*P* = 0.013) [25]. Kudo et al. reported that adding HAIC with 5-FU and cisplatin to sorafenib might improve overall survival in HCC patients with main portal vein invasion (11.4 vs. 6.5 months) [26]. While its benefits have not been confirmed in a randomized control study, HAIC with 5-FU and cisplatin may offer a better response to treatment than sorafenib in advanced HCC patients with massive macrovascular invasion.

It is controversial whether duration of HAIC reflects therapeutic effect. In the previous reports that described pathological CR by HAIC alone, the treatment periods ranged from 3 to 26 months [17–20]. In the present case, tumor progression was observed after regression following a single cycle of initial HAIC, which suggests that the treatment period was too short. Following long-term HAIC for 21 months would control the tumor and lead to the complete remission. Based on the fact that the present HCC showed a hypovascular appearance, tumor vascularity might also relate to the tumor shrinkage. HCC tends to appear hypovascular and heterogeneous on contrast-enhanced CT if an HCC patient has a high level of serum vascular endothelial growth factor (VEGF) [27]. Abnormal tumor vascular networks induced by VEGF develop tumor hypoxia: an important factor of spontaneous tumor regression [28, 29]. Thus, hypovascular appearance as well as long-term HAIC would contribute to the complete remission in the present case. Prognosis of non-responders to HAIC was known to be poor, and remarkable responses as in the present case are rare and challenging. Therefore, establishment of a pretherapeutic assessment of candidates for HAIC is needed to provide optimal treatment to patients with advanced HCC.

## Conclusion

Even though only systemic chemotherapy has been approved worldwide for patients with advanced unresectable HCC, the present case suggests HAIC has been effective and can be an alternative treatment option for advanced HCC with macrovascular invasion.

## Data Availability

All data generated or analyzed in the current article are available from the corresponding author on reasonable request.

## Abbreviations

5-FU: 5-Fluorouracil
AFP: Alpha-fetoprotein
CT: Computed tomography
HAIC: Hepatic arterial infusion chemotherapy
HCC: Hepatocellular carcinoma
IVCTT: Inferior vena cava tumor thrombosis
MST: Median survival time
PIVKA-II: Protein induced by vitamin K absence or antagonist-II
PVTT: Portal vein tumor thrombosis
